# Revisiting alkaline cupric oxide oxidation method for lignin structural analysis

**DOI:** 10.3389/fbioe.2022.1002145

**Published:** 2022-09-07

**Authors:** Guangxu Yang, Zhenggang Gong, Xiaolin Luo, Li Shuai

**Affiliations:** College of Materials Engineering, Fujian Agriculture and Forestry University, Fuzhou, China

**Keywords:** lignin, depolymerization, oxidation, cupric oxide, model compounds

## Abstract

Lignin structural analysis is important for the comprehensive utilization of lignin as well as delignification and bleaching during pulping while it is difficult to completely elucidate lignin structure due to its structural complexity and heterogeneity. Depolymerization of lignin into simple monomers via alkaline cupric oxide oxidation (Ox^CuO^) followed by chromatographic analysis of the monomers is an effective method for lignin structural analysis. Here we revisited the Ox^CuO^ of lignin model compounds (monomers and dimers) and three representative lignocelluloses (i.e., Eucalyptus, Masson pine, and corn stover) to understand the effects of reaction conditions and lignin sub-structures on oxidation product yields and distributions. The improved Ox^CuO^ was found to be effective in oxidatively breaking the robust interunit C-C bonds in the β-β′ and β-5′ moieties of lignin other than β-O-4′ linkages at an elevated temperature (210°C). Further degradation of the monomeric oxidation products could also occur to reduce the monomer yields under a severe condition (i.e., high temperature and long reaction time). In addition, O_2_ inputs could reduce the monomer yields via nonselective overoxidation, thus having negative effects on accurate structural analysis of lignin. The O_2_ removal via ultrasonication combined with N_2_ flushing prior to the oxidation reaction could improve the monomer yield about 1.2 times (compared to that without O_2_ removal) at a low biomass loading of 5 wt%. By using the improved method of Ox^CuO^, a monomer yield of 71.9% could be achieved from Eucalyptus (hardwood) lignin, which was much higher than conventional nitrobenzene oxidation (59.8%) and reductive depolymerization (51.9%). Considering the low cost, high availability, and low toxicity of CuO, the improved Ox^CuO^ could be a convenient and economic method for more accurate lignin structural analysis.

## Introduction

**FIGURE 1:**
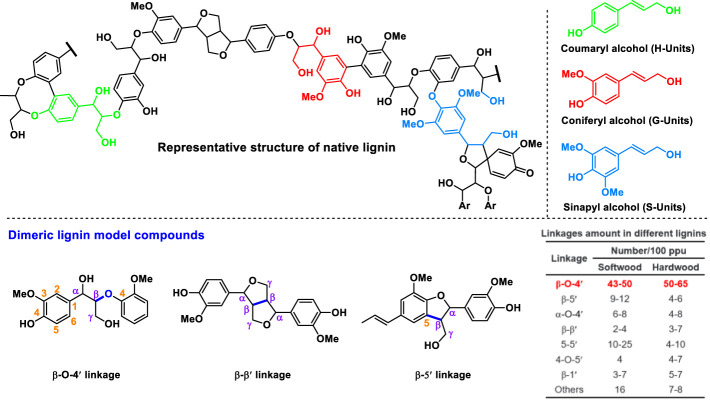
The representative structure of native lignin and the dimeric lignin model compounds used in this study.

Lignin is synthesized by oxidative radical polymerization of syringyl (S), guaiacyl (G), and p-hydroxyphenyl (H) subunits, and the monomeric units are connected by several types of C-O and C-C linkages (Figure 1) (Deussa and Barta., 2016; Zhang and Wang., 2020). As one of the main component of lignocellulosic biomass (Schutyser et al., 2018), structural analysis of lignin is of great importance for its comprehensive utilization as well as delignification and bleaching during pulping. However, comprehensive analysis of lignin structure is challenging due to its complex and heterogeneous structure. To obtain lignin structural information, depolymerization (or degradation) of lignin into simple lignin monomers followed by chromatographic analysis is the most convenient and effective method. Many methods such as acidolysis (Voitl and Rohr., 2008; Partenheimer, 2009; Werhan et al., 2011), thioacetolysis (Nimz, 1974), and reductive depolymerization (Lu and Ralph., 1997; Bosch et al., 2015; Shuai et al., 2016; Liao et al., 2020) have been developed to evaluate the content and structure of lignin in lignocellulosic materials. However, these methods have certain drawbacks such as inability to break the C-C bonds, long analysis time, and/or costly catalysts or reagents. For example, oxidative depolymerization of lignin using an oxidant (e.g., nitrobenzene) in an alkaline solution (Ox^NB^) is effective in cleavage of both C-O and C-C bonds to give a higher lignin monomers yield (Chan et al., 1995; Hirayam et al., 2019), while nitrobenzene is a toxic compound and produces many by-products that can interfere with the analysis of lignin monomers (Lapierre, 2010). In contrast, cupric oxide (CuO) as a common oxidant is more competitive than nitrobenzene due to its low cost, high availability, and low toxicity. Furthermore, CuO and its reduction products (Cu or Cu_2_O) have very little interference with the analysis of lignin monomers. CuO as a heterogeneous catalyst has poor solubility in NaOH aqueous solution, thus facilitating the isolation of the catalyst and liquid products via simple centrifugation (Chen, 1992; Lapierre, 2010).

Previously, a reported method of alkaline cupric oxide oxidation (Ox^CuO^) for lignin structural analysis could achieve a phenolic monomer yield of around 50.7 mol% from native hardwood lignin and the monomers were believed to be resultant from the selective cleavage of C-O interunit linkages such as β-O-4 linkage (Chen, 1992). Since the structure of the aromatic nuclei (i.e., guaiacyl, syringyl, and 4-hydroxyphenyl structures) on lignin are preserved after Ox^CuO^, Ox^CuO^ can give an indication of the composition of lignin units under investigation, and the yields of the oxidation products can provide information on the composition of lignin interunit linkages (Hedges and Ertel., 1982; Goi et al., 1993). For example, hardwoods such as *Eucalyptus* or birch gave both guaiacyl and syringyl products while softwood only gave guaiacyl products. 4-hydroxyphenyl products are generally observed in the oxidation of grass together with guaiacyl and syringyl products (Schutyser et al., 2018). As for lignin with highly condensed structures, little monomeric oxidation products can be produced (Billa et al., 1996; Villar et al., 1997). Based on the differences of the monomeric oxidation products, Ox^CuO^ can be used to identify biomass species (hardwood, softwood, or grass) and analyze unknown biomass samples.

Recently, we revisited the method of Ox^CuO^ and found that three improved aspects would facilitate more accurate and efficient analysis of lignin structures. First, an elevated temperature and a prolonged reaction time facilitated the cleavage of C-C bonds (e.g., β-β′ and β-5′) in lignin during Ox^CuO^ of lignin while it could also lead to increased degradation of the oxidation products. To obtain high-yield lignin monomers, an optimized reaction condition should be explored to enable the cleavage of C-C bonds meanwhile minimizing the degradation of the desired products. Second, the operation of O_2_ removal before oxidative depolymerization was necessary to avoid the overoxidation of the monomeric oxidation products, which could improve the monomer yields especially at a low biomass loading. Choosing a simpler method (such as ultrasonication combined with N_2_ flushing) to effectively remove O_2_ could shorten the analysis time compared to the thermal or chemical oxygen removal. Third, for analysis of the lignin monomers in alkaline aqueous solution after oxidative depolymerization, the classical procedures involved acidification of the hydrolysate, followed by the extraction of phenolic products for HPLC or GC analysis (Chen, 1992; Lapierre, 2010; Tamai et al., 2015; Schutyser et al., 2018). The possibility of the incomplete extraction could result in an underrated yield of lignin monomers and poor reproducibility. Therefore, an improved product analysis method should be developed to determine all lignin monomers in the hydrolysate. These thoughts motivate us to develop an improved method of alkaline cupric oxide oxidation for more accurate and readily lignin structural analysis.

## Materials and methods

### Materials

Chemicals including methylguaiacol (2-methoxy-4-Methylphenol, >98%), ethylguaiacol (2-methoxy-4-ethylphenol, 99%), propylguaiacol (2-methoxy-4-propylphenol, 98%), *p*-methylphenol (99%), methylsyringol (2,6-dimethoxy-4-methylphenol, >97%), vanillin (4-hydroxy-3-methoxybenzaldehyde, 99%), acetovanillone (4′-hydroxy-3′-methoxyacetophenone, 98%), vanillic acid (4-hydroxy-3-methoxybenzoic acid, 98%), syringaldehyde (4-hydroxy-3,5-dimethoxybenzaldehyde, 98%), acetosyringone (4′-hydroxy-3′,5′-dimethoxyacetophenone, 98%), syringic acid (4-hydroxy-3,5-dimethoxybenzoic acid, 98%), nitrobenzene (≥99%), CuO (40 nm particle size, 99.5%), benzoic acid (≥99.9%), anhydrous pyridine (≥99%), Ru/C catalysts (5% metal loadings), and NaOH (≥98%) were purchased from Aladdin^®^ Biochemical Technology Co., Ltd. (Shanghai, China). BSTFA (N, O-bis(trimethylsilyl)trifluoroacetamide, >99%) and hydrochloric acid (37%) were purchased from Sigma Aldrich (Shanghai, China). Guaiacylglycerol-β-guaiacyl ether [1-(4-hydroxy-3-methoxyphenyl)-2-(2-methoxyphenoxy)propane-1,3-diol, 97%] and pinoresinol [4-[(3S,3aR,6S,6aR)-6-(4-hydroxy-3-methoxyphenyl)-1,3,3a,4,6,6a-hexahydrofuro [3,4-c]furan-3-yl]-2-methoxyphenol, 98%] were purchased from TCI Chemicals (Shanghai, China). Dehydrodiisoeugenol (4-(2,3-dihydro-7-methoxy-3-methyl-5-propenyl-2-benzofuranyl)-2-methoxyphenol, 98%) were purchased from Yuanye Bio-Technology Co., Ltd. (Shanghai, China). All commercial chemicals were analytical reagents and were used as received. Water was purified using a Millipore Milli-Q I water purification system to a resistivity higher than 18 MΩ cm.

Corn stover was provided by the State Key Laboratory of Biobased Materials and Green Papermaking at Qilu University of Technology, China. Masson pine and Eucalyptus wood chips were provided by Fujian Qingshan Paper Co., Ltd. (Sanming, China). Wood chips and corn stover were air-dried, carefully milled to pass through a screen of 40 mesh for experiments.

### Reductive depolymerization

In the typical reductive depolymerization experiments, 500 mg of air-dried wood particles (>40 mesh) (or 50 mg of dimeric lignin model compounds), 100 mg of Ru/C (5 wt% Ru on Carbon) and 10 ml of methanol were loaded into a 25-ml pressure-resistant reactor. The reactor was closed, purged three times with H_2_ and pressurized with 4 MPa H_2_. The mixture was stirred with a magnetic bar at 800 rpm, heated with a heating jacket controlled by a PID temperature controller to 220°C and then held at the temperature for 10 h. After the reaction, the reactor was cooled with tap water. One milliliter of internal standard solution (30 mg/ml benzoic acid in dioxane) was directly added into the reactor and mixed with the slurry. One milliliter of the clear solution was sampled and centrifuged. The resultant supernatant was used for GC or GC-MS analysis.

### Alkaline nitrobenzene oxidation

Nitrobenzene oxidation of lignins and dimeric lignin model compounds were performed in a 25-ml pressure-resistant reactor. Specifically, 7 ml of 2 mol/L NaOH aqueous solution, 200 mg of air-dried wood particles (>40 mesh) (or 50 mg of dimeric lignin model compounds) and 0.4 ml of nitrobenzene were added to the reactor. The reactor was pressurized with N_2_ to 0.2 MPa. Then the reactor was heated to 170°C and held at the temperature for 2.5 h. After cooling, the reaction mixture in the reactor was transferred to a 100-ml separating funnel and extracted by 25 ml of chloroform three times to remove the residual nitrobenzene and its derivatives. The aqueous phase was acidified by concentrated hydrochloric acid to pH < 3. The acidified liquid was extracted with fresh chloroform (3 × 25 ml) and the chloroform phases were combined. One milliliter of internal standard solution (30 mg/ml benzoic acid in dioxane) was mixed with the chloroform phases. The resultant liquid was used for GC and GC-MS analyses.

### Alkaline copper oxide oxidation

For the alkaline copper oxide oxidation experiments, 1 g of air-dried wood particles (>40 mesh) (or 50 mg of dimeric lignin model compounds) and 1.5 g of CuO (40 nm) were mixed with 10 ml of NaOH aqueous solution (2.5 mol/L) in a 25-ml pressure-resistant reactor. The mixture was ultrasonically treated for 10 min and the reactor was sealed, flushed with N_2_ three times, and then pressurized with N_2_ to 0.2 MPa. The reactor was heated in a heating jacket to 210°C and maintained at the temperature for 40 min. The reaction was stirred with a magnetic bar at 800 rpm. After the reaction, the reactor was quickly cooled to room temperature with tap water. Due to its good solubility in both alkaline solution and organic solvent, 1 ml of benzoic acid standard solution (benzoic acid dissolved in dioxane with a concentration of 30 mg/ml) was directly added into the reactor and mixed with the slurry. One milliliter of the clear solution was sampled and centrifuged. The resultant supernatant was neutralized and used for GC or GC-MS analysis.

In order to investigate the effect of O_2_ on lignin monomer yields, the operation of O_2_ removal was not required for the original mixtures. Alternatively, these reactors were either just pressurized with N_2_ to 0.2 MPa or directly pressurized with O_2_ to the specified pressure. The other experimental procedures were the same as that mentioned above.

### Gas chromatography and gas chromatography-mass spectrometry analyses

Lignin monomers resulted from the alkaline copper oxide oxidation were initially identified by GC-MS and then quantified by GC. Prior to the GC and GC-MS analysis, the sample was derived with BSTFA. Specifically, 10 μl of the sample solution and 5 μl of concentrated HCl (37 wt%) were mixed with 200 μl of pyridine in a 2-ml GC vial. After ultrasonic treatment for 30 s, 700 μl of BSTFA was added to the GC vial which was then kept at 80°C for 1 h. The silylated products were identified by GC-MS using an Agilent 7890B series GC equipped with a HP5-MS capillary column (30 m × 0.45 mm) and an Agilent 5977A series mass spectroscopy detector. The inlet and detector temperature were 300°C, and the injection volume was 1 μl. Helium was used as a carrier gas at a flow rate of 1.5 ml/min. The column was initially kept at 50°C for 5 min, then was heated at a rate of 10°C/min to 300°C and held for 5 min. Most of the products were directly identified with authentic standard compounds. Some of products were directly identified according to the mass spectra. As for the lignin monomers from reductive depolymerization and alkaline nitrobenzene oxidation (chloroform phases), the samples were completely processed with the method used for alkaline copper oxide oxidation, except that the concentrated HCl was not required to add.

The identified products were further quantified by a GC (Agilent 7890B series, United States) equipped with an HP5 capillary column and a flame ionization detector (FID). The yields of the products in the sample solution were calculated based on the effective carbon number rule for convenience. The detailed calculation was as follows:

In the equations,

$${\text{n}}_{product}=n_{benzoic\;acid}\times \frac{A_{product\;}}{A_{benzoic\;acid}}\times \frac{ECN_{benzoic\;acid}}{ECN_{product}}$$ (1)

$$Y_{product}=\frac{n_{product}}{n_{theoretical}}$$ (2)

*n*_*benzoic acid*_ (mmol): the molar amount of the internal standard (benzoic acid); *n*_*product*_ (mmol): the molar amount of the lignin monomers; *A*_*product*_: the peak area of monomers in the GC-FID chromatogram; *A*_*benzoic acid*_: the peak area of benzoic acid in the GC-FID chromatogram; *ECN*_*benzoic acid*_: the effective carbon number of silylated benzoic acid (Shuai et al., 2016); *ECN*_*product*_: the effective carbon number of the lignin monomers; *Y*_*product*_: 1) For the depolymerization of lignin model compounds (monomers and dimers), the molar yield of monomers was calculated on the basis of the molar amount of aromatic rings in the model compounds (n_theoretical_); 2) for the depolymerization of lignin, the molar yield of monomers was calculated on the basis of the molar amount of Klason lignin (n_theoretical_); an average molecular weight of 210 g/mol (Chen, 1992) for lignin monomeric units was used for the calculations.

## Results and discussion

### Alkaline copper oxide oxidation of lignin model compounds

#### Monomeric lignin model compounds

**FIGURE 2:**
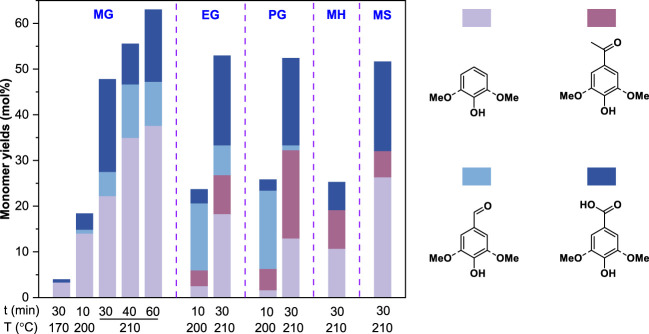
Alkaline copper oxide oxidation of monomeric lignin model compounds. MG, EG, PG, MH, and MS represented methylguaiacol, ethylguaiacol, propylguaiacol, *p*-methylphenol, and methylsyringol a respectively. The model compound loadings were 50 mg. All reactions were conducted in 10 ml of 2.5 mol/L NaOH with 2 g CuO addition.

Several monomeric lignin model compounds with different alkyl side chains (i.e., methylguaiacol, MG; ethylguaiacol, EG; propylguaiacol, PG) that are typically produced from reductive depolymerization (RD) were oxidized with CuO in an alkaline condition. After the reaction, the products were analyzed by gas chromatography with flame ionization detection or mass spectrometry (GC-FID and GC-MS) *via* an improved method as described in the METHODS. The results in Figure 2 suggested that alkaline copper oxide oxidation (Ox^CuO^) could even break the alkyl side chains (or C-C bonds) of lignin monomers to give oxidation products (e.g., vanillin, vanillic acid, and acetovanillone) at 200°C for 10 min. In contrast, almost no monomeric oxidation products were observed after the Ox^CuO^ of MG (<5%) under a conventional condition (170°C, 30 min) (Chen, 1992), while the yields of the monomeric oxidation products for MG, EG and PG were all significantly increased about two times under a severe condition (210°C, 30 min). Prolongation of the reaction time further improved the yields of the monomeric oxidation products for MG from 47.8% (30 min) to 55.6% (40 min) and 63.1% (60 min), respectively. These results indicated that an elevated temperature and a long reaction time facilitated the C-C cleavage during Ox^CuO^. Similar monomeric oxidation products were also observed after the Ox^CuO^ of *p*-methylphenol (MH) and methylsyringol (MS) that had different aromatic nuclei. In addition, an H-type (MH, 25.3%) model gave oxidation products in much lower yield than G-type (MG, 47.8%) and S-type (MS, 51.7%) models (Figure 2). It was in line with the result of nitrobenzene oxidation (Chan et al., 1995), where electron donating groups (i.e., methoxyl groups) on the aromatic nuclei of lignin monomers enhanced the rate of oxidation. Therefore, the structure of the aromatic nucleus could also affect the reactivity of these lignin monomers to give oxidation products apart from the reaction temperature and time, which could be explained by the different ability of the aromatic nuclei to supply electrons to the benzylic carbon atoms (i.e., syringyl > guaiacyl > 4-hydroxyphenyl) (Shuai and Saha., 2017).

#### Dimeric lignin model compounds

Considering the good performance of Ox^CuO^ on breaking C-C bonds in lignin monomers, we further examined its ability to oxidatively break the robust interunit C-C bonds in the β-β′ and β-5′ moieties of lignin other than β-O-4′ linkages. Three representative dimeric lignin model compounds present in Figure 1 were selected as substrates for oxidative depolymerization (Ox^CuO^ and Ox^NB^).

**FIGURE 3:**
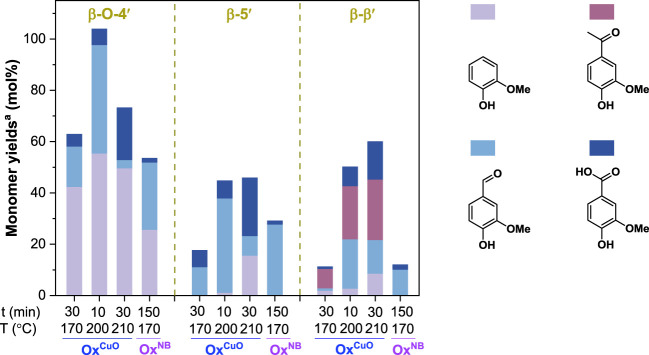
Alkaline oxidative depolymerization of dimeric lignin model compounds with CuO or nitrobenzene as the oxidant. ^a^The monomer yields were based on the molar amount of aromatic rings in the model compounds. The model compound loadings were 50 mg. All CuO oxidation experiments were conducted in 10 ml of 2.5 mol/L NaOH with 0.5 g CuO addition. All nitrobenzene oxidation experiments were conducted in 7 ml of 2.0 mol/L NaOH with 0.4 ml nitrobenzene addition.

As showed in Figure 3, the β-O-4′ dimeric compound gave a higher monomer yield (62.9%) for Ox^CuO^ at 170°C for 30 min than Ox^NB^ at 170°C for 2.5 h (53.5%). As the reaction condition was intensified, the monomer yield for Ox^CuO^ of the β-O-4′ dimeric compound increased first and then decreased, and a theoretical monomer yield was achieved at 200°C for 10 min. The high monomer yield suggested that no condensation reaction of the monomeric products occurred in such Ox^CuO^ system. The decreased monomers under a severe condition (210°C for 30 min) could be due to the further degradation of the monomer products. Nevertheless, such severe condition facilitated the cleavage of C-C bonds in β-5′ and β-β′ dimeric compounds. The highest monomer yields of 44.8% and 50.2% were achieve at 210°C for 30 min via oxidatively breaking β-5′ and β-β′ bonds, respectively. These results suggested that an elevated temperature (210°C) was necessary for β-5′ and β-β′ cleavage, which was in line with the results of oxidation of alkyl side chains in lignin monomers discussed above.

#### Stability tests of monomeric oxidation products

**FIGURE 4:**
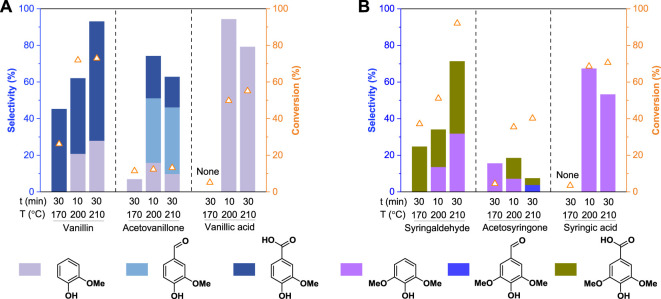
Stability tests of **(A)** G-type and **(B)** S-type monomeric oxidation products in the alkaline copper oxide oxidation system. The monomeric oxidation products loadings were 50 mg. All oxidation experiments were conducted in 10 ml of 2.5 mol/L NaOH with 0.5 g CuO addition.

As discussed above, the Ox^CuO^ system could not only enable the oxidative depolymerization of lignin, but also could reduce the monomer yields via further oxidative degradation of the oxidation products. To investigate the stability of the oxidation products, we performed the Ox^CuO^ of both G-type and S-type oxidation products under different conditions. The conversion of the substrates and the selectivity of the oxidation products were presented in Figure 4. For example, vanillin could readily be converted to vanillic acid at a relatively low temperature (170°C), guaiacol was only produced from vanillin at an elevated temperature (>200°C). Although the formed vanillic acid showed good stability at 170°C, substantial conversion of vanillic acid (49.8%) and high guaiacol selectivity (94.4%) could be achieved at 200°C for 10 min (Figure 4A). These results suggested that once vanillin was formed in the Ox^CuO^ system, it could be readily oxidized to vanillic acid, followed by the formation of guaiacol via further decarboxylation at an elevated temperature (>200°C). Whereas only 13.2% of the acetovanillone was further converted to other monomer products (i.e., guaiacol, vanillin, and vanillic acid) with a total selectivity of 62.9% even under a severe condition (210°C for 30 min), confirming that acetovanillone was relatively stable in the Ox^CuO^ system. As for S-type monomeric oxidation products, a transformation path similar to G-type monomeric oxidation products was observed. However, compared to G-type monomers, S-type monomers showed higher reactivity (or conversion rate) and were more easily degraded (Figure 4B), which was consistent with the result that S-type alkylphenols had the highest oxidation reactivity (Figure 2). The above results proved that the poor stability of the monomeric oxidation products under a severe oxidation condition was the main reason for the loss of monomer yields during oxidative degradation of lignin (or lignin model compounds). Therefore, the optimal temperatures for the oxidative cleavage of different interunit linkages should be carefully evaluated for the high-yield monomer production and more accurate structural analysis.

### Alkaline copper oxide oxidation of native lignin

#### The effect of oxygen inputs on monomer yields

**FIGURE 5:**
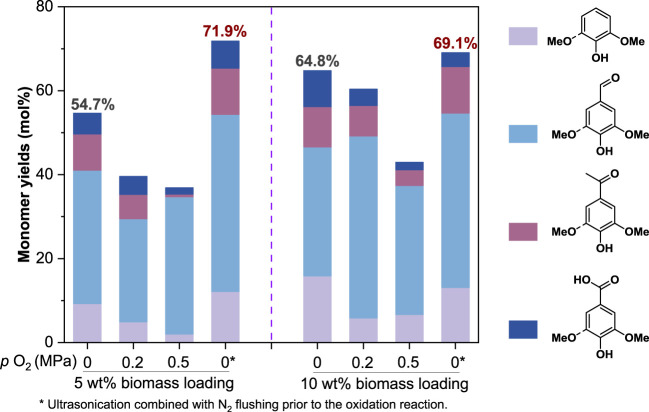
The effect of oxygen inputs on lignin monomer yields (on lignin basis). All reactions were conducted at 210°C for 40 min with 1.5 g CuO and 10 ml of 2.5 mol/L NaOH addition.

To examine the performance of Ox^CuO^ on lignin depolymerization, we conducted the reactions with Eucalyptus wood particles. By following the traditional procedure (Chen, 1992), a monomer yield of only 54.7% was achieved at a 5 wt% biomass loading of 5 wt% (Figure 5). However, the previous model compound studies have shown that the Ox^CuO^ enabled the cleavage of β-O-4′, β-β′, and β-5′, a theoretical monomer yield (based on the total content of β-O-4′, β-β′, and β-5′ moieties of hardwood lignin, Figure 1) could be around 70% if all of these linkages were selectively cleaved. Such difference (54.7% vs. 70%) motived us to explore the possible factors that caused the lowered oxidation product yield and to provide a more accurate method for lignin structural analysis.

Since O_2_ was known to facilitate both product formation and degradation during alkaline aerobic lignin oxidation (Mathias and Rodrigues., 1995; Wu and Heitz., 1995; Schutyser et al., 2018), we speculated that the dissolved O_2_ in the reaction liquid and residual O_2_ in the reactor might be responsible for the low monomer yield especially at a low biomass loading. To validate the speculation, we inspected the lignin monomer yields for Ox^CuO^ of Eucalyptus wood particles with various O_2_ inputs. As expected, even a 0.2 MPa O_2_ input could significantly reduce the monomer yields from 54.7% to 39.6% at a relatively low biomass loading of 5 wt% (Figure 5), suggesting that the existence of O_2_ had negative effect on the Ox^CuO^ process. While further increasing the O_2_ input to 0.5 MPa had a slight effect on the monomer yield (36.9%) due to the limited reaction time. Interestingly, an improved monomer yield to 64.8% was observed when the biomass loading was increased to 10 wt%. It was because the high biomass loading mitigated the negative effect of the side reactions (such as overoxidation) on the monomer production in the reactor where a fixed volume of oxygen was present. Under the condition of a limited amount of O_2_ input (<0.2 MPa) and a high biomass loading of 10 wt%, a comparable monomer yield (60.4%) to the condition of no extra O_2_ input (64.8%) could be obtained. When the reactor was pressurized with up to 0.5 MPa O_2_, the monomer yield was dramatically reduced to below 50%. Therefore, the existence of O_2_ was an obstacle towards accurate analysis of lignin structure. In order to obtain high yields of monomers, it was preferable to remove O_2_ from the reaction liquid and the reactor via ultrasonication combined with N_2_ flushing prior to the oxidation reaction (see METHODS); besides, higher biomass loading could mitigate the effect of O_2_ on monomer yields. Monomer yields of 71.9% and 69.1% could be achieved via the improved method of Ox^CuO^ at a biomass loading of 5 wt% and 10 wt%, respectively, which were close to the theoretical monomer yield (around 70%).

#### Comparison of different methods for lignin structural analysis

The types and ratios of subunits as well as the amount of interunit linkages in lignin vary with the types and sources of plants (Vanholme et al., 2010; Maeda, 2016; Tolbert et al., 2016). Therefore, three representative lignocelluloses (i.e., *Eucalyptus*, Masson pine, and corn stover) were selected as feedstocks to study the depolymerization of lignin in hardwood, softwood, and grass, respectively. The oxidative degradation of lignin was conducted in NaOH aqueous solution with CuO or nitrobenzene as an oxidant. Classical reductive depolymerization of lignin was conducted in methanol with Ru/C as a catalyst.

**FIGURE 6:**
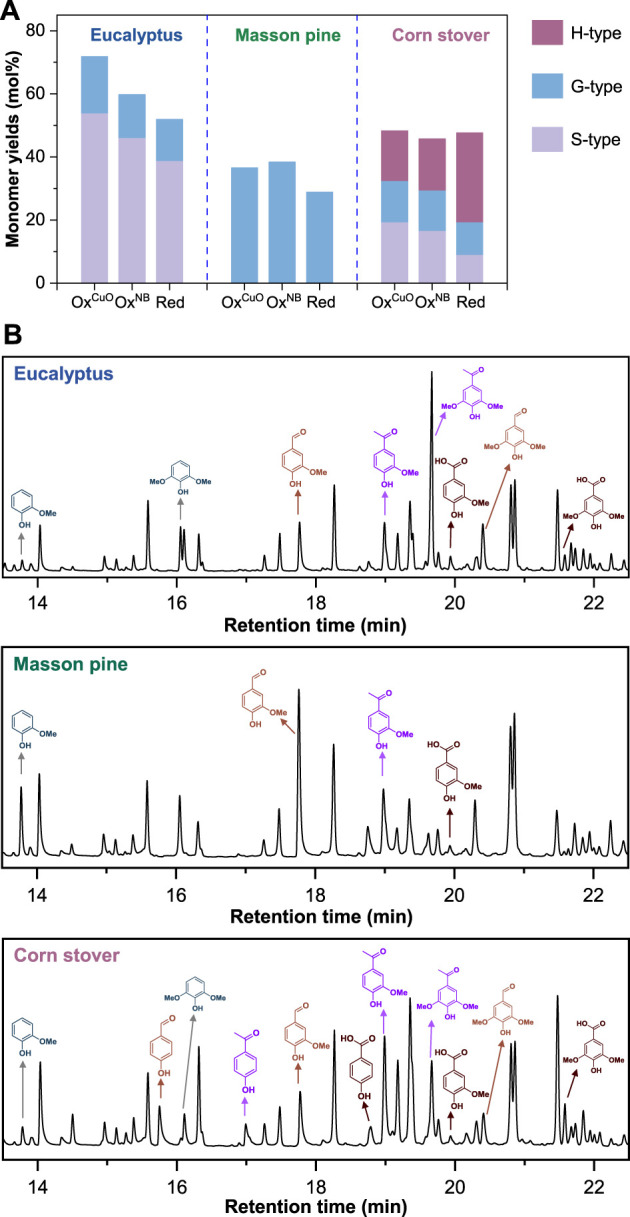
**(A)** Comparison of lignocellulose alkaline copper oxide oxidation with conventional nitrobenzene oxidation and reductive depolymerization. **(B)** Gas chromatograms of the lignin monomers derived from alkaline copper oxide oxidation of lignin. The detailed reaction conditions were described in *Materials and Methods*.

As shown in Figure 6A, reductive depolymerization (Red) of lignin enabled monomer yields of 51.9% for Eucalyptus lignin, 28.9% for Masson pine lignin, 47.6% for corn stover lignin via the cleavage of only labile C-O ether linkages (Bosch et al., 2015; Shuai et al., 2016). In contrast, higher yields of lignin monomers could be obtained *via* oxidative depolymerization due to the additional cleavage of C-C bonds. The total monomer yield of 71.9% for Ox^CuO^ of Eucalyptus was much higher than that of 59.8% for Ox^NB^ and 51.9% for Red, demonstrating that the improved Ox^CuO^ had better performance (shorter reaction time and higher monomer yields) on lignin depolymerization than conventional Red and Ox^NB^. Although the similar products could be obtained from the three lignocelluloses (Figure 6B), the least monomers yield were provided by Masson pine using the same oxidant (CuO or nitrobenzene) (Figure 6A). Furthermore, the contents of condensed structures such as biphenyl structures (5–5′ linkages) in softwood lignin (10%–25%, Figure 1) were higher than those in hardwood lignin (4%–10%). Such 5-5′ bonds were unable to be broke by such oxidation system (Tamai et al., 2015), limiting the release of monomers. Therefore, the improved method of Ox^CuO^ could increase the lignin monomer yields of Eucalyptus more obviously than Masson pine and corn stover.

## Conclusion

The results of the lignin model compound experiments coupled with the chromatographic analysis provide new understanding towards the alkaline copper oxide oxidation of lignin (Ox^CuO^). First, we found that other than β-O-4′ moieties of lignin, the β-β′ and β-5′ moieties of lignin also contributed to the monomeric oxidation products via the cleavage of C-C interunit linkages even under the classical Ox^CuO^ conditions (170°C, 30 min). Therefore, compared to the classical Ox^CuO^ method which ascribed lignin monomers to the cleavage of C-O ether linkages, the improved Ox^CuO^ method could be more accurate for characterizing the structure of native lignins. Second, the direct addition of benzoic acid as an internal standard in the alkaline solution combined with the direct silylation of the reaction liquor simplified the product analysis procedure, which further improved the accuracy of the improved Ox^CuO^ method for analyzing lignin structure. Third, high temperature and O_2_ removal were desirable for the high-yield production of lignin monomers from lignocelluloses via Ox^CuO^. While the elevated temperature not only increased the degree of lignin depolymerization but also promoted the degradation of the monomeric oxidation products. By optimizing the reaction condition of Ox^CuO^ and the procedure for products analysis, an improved monomer yield of 71.9% could be achieved from Eucalyptus wood particles. This result was better than the classical Ox^CuO^ (54.7%), the conventional nitrobenzene oxidation (59.8%) and reductive depolymerization (51.9%). Since all of the three moieties (β-O-4′, β-β′, and β-5′) of lignin could produce similar monomeric oxidation products *via* Ox^CuO^, it was difficult to distinguish the source of oxidation products. However, the amount of non-condensed units in lignin could be estimated according to the lignin monomers yield from the reductive depolymerization of lignin while the enhanced lignin monomers yield from the oxidative depolymerization of lignin could be used to estimate the content of breakable C-C interunit linkages (i.e., β-β′ and β-5′) in lignin. Therefore, the improved Ox^CuO^ could be a more applicable method for fast and accurate analysis of lignin non-condensed units and condensed units, estimation of the monomer yields for lignin depolymerization, and comparison of the condensation degree of lignin polymers.

## Data Availability

The original contributions presented in the study are included in the article/supplementary material, further inquiries can be directed to the corresponding authors.
